# INTRApersonal Synchrony as Constituent of INTERpersonal Synchrony and Its Relevance for Autism Spectrum Disorder

**DOI:** 10.3389/frobt.2019.00073

**Published:** 2019-08-20

**Authors:** Carola Bloch, Kai Vogeley, Alexandra L. Georgescu, Christine M. Falter-Wagner

**Affiliations:** ^1^Department of Psychiatry and Psychotherapy, Medical Faculty, University of Cologne, Cologne, Germany; ^2^Department of Psychiatry and Psychotherapy, Medical Faculty, Ludwig-Maximilians-University, Munich, Germany; ^3^Institute of Neuroscience and Medicine (INM3), Research Center Jülich, Jülich, Germany; ^4^Department of Psychology, Institute of Psychiatry, Psychology and Neuroscience, King's College London, London, United Kingdom; ^5^Department of Psychology, Faculty of Human Science, University of Cologne, Cologne, Germany

**Keywords:** human-human interaction, INTERpersonal synchrony, INTRApersonal synchrony, timing, non-verbal behavior, autism spectrum disorder

## Abstract

INTERpersonal synchrony leads to increased empathy, rapport and understanding, enabling successful human-human interactions and reciprocal bonding. Research shows that individuals with Autism Spectrum Disorder (ASD) exhibit difficulties to INTERpersonally synchronize but underlying causes are yet unknown. In order to successfully synchronize with others, INTRApersonal synchronization of communicative signals appears to be a necessary prerequisite. We understand INTRApersonal synchrony as an implicit factor of INTERpersonal synchrony and therefore hypothesize that atypicalities of INTRApersonal synchrony may add to INTERpersonal synchrony problems in ASD and their interaction partners. In this perspective article, we first review evidence for INTERpersonal dissynchrony in ASD, with respect to different approaches and assessment methods. Second, we draft a theoretical conceptualization of INTRApersonal dissynchrony in ASD based on a temporal model of human interaction. We will outline literature indicating INTRApersonal dissynchrony in ASD, therefore highlighting findings of atypical timing functions and findings from clinical and behavioral studies that indicate peculiar motion patterns and communicative signal production in ASD. Third, we hypothesize that findings from these domains suggest an assessment and investigation of temporal parameters of social behavior in individuals with ASD. We will further propose specific goals of empirical approaches on INTRApersonal dissynchrony. Finally we present implications of research on INTRApersonal timing in ASD for diagnostic and therapeutic purposes, what in our opinion warrants the increase of research efforts in this domain.

## 1. INTERpersonal Dissynchrony

“*Terms such as interactional synchrony, non-verbal mirroring, shared rhythmicity, motor mimicry or chameleon effect embrace the underlying dimension of coordination between two or more individuals in the domain of nonverbal action*” (Ramseyer and Tschacher, 2008, p.332).

Across different terminology INTERpersonal synchrony describes the phenomenon that people automatically align behavior while interacting. This is thought to strengthen their social bond by means of increased rapport (LaFrance, 1979; Tickle-Degnen and Rosenthal, 1990; Lakin and Chartrand, 2003; Vacharkulksemsuk and Fredrickson, 2012), mutual affiliation (Hove and Risen, 2009), enhanced mentalizing (Baimel et al., 2018), successful joint action (Valdesolo et al., 2010; Lorenz et al., 2014), as well as empathy (Behrends et al., 2012; Koehne et al., 2016). Autism Spectrum Disorder (ASD) is defined as a neurodevelopmental disorder that entails difficulties in social communication and interaction together with repetitive behaviors and restricted interests (American Psychiatric Association, 2013) and there exists evidence for INTERpersonal dissynchrony of individuals with ASD with interaction partners.

INTERpersonal synchrony as a dependent variable in groups of healthy control persons was measured in reference to parameters in a dynamical model of human movements dynamical model of human movements (Haken et al., 1985). Those studies measured coordinated movements between two individuals in terms of reduced changes in relative phase angles between reference points in two oscillating systems (Richardson et al., 2007; Schmidt and Richardson, 2008; Romero et al., 2015). With respect to individuals with ASD one study found less coordination of movements measured by the alignment of phase angles between two rocking chairs (Marsh et al., 2013). Similarly, individuals with ASD synchronized pendulum swings less with their parents (Fitzpatrick et al., 2016).

Fitzpatrick et al. (2017a) separately investigated performance in intentional vs. spontaneous synchrony tasks and found lower INTERpersonal coherence scores in both domains for children with ASD. Additionally, the authors found distinct cognitive mechanisms underlying both kinds of alignment problems (Fitzpatrick et al., 2017b). In a naturalistic setting, spontaneous INTERpersonal synchrony was measured by coherence of body motion of two interaction partners in predefined regions of interest, thereby not focusing on external oscillators or specific limbs, rather on general body motion (Ramseyer and Tschacher, 2011; Romero et al., 2015). So-called Motion Energy Analysis (MEA) (Ramseyer and Tschacher, 2011) calculates cross-correlation time series of pixel changes from video-recorded interactions as an indicator for coordinated movements. Noel et al. (2018) used MEA in their study and showed that individuals with ASD exhibited less INTERpersonal synchrony and less complex movements in an interview setting.

Relevant for understanding INTERpersonal synchrony are also joint action paradigms, in which participants have to conduct actions that require consideration of another person's perspective and movement affordances in the course of motion planning. When assessing the motor anticipation of a partner's grip comfort when passing objects, participants with ASD showed more variable grip positions indicating atypical social motor planning (Gonzalez et al., 2013). Moreover, with increasing severity of ASD traits, participants modulated grip movements less in adaption to a partner's movements, but performed well in a non-social replication task indicating deficits only for the social domain (Curioni et al., 2017). Other studies found less grip-to-reach positions that enhanced end-state comfort for the partner (Scharoun and Bryden, 2016; Studenka et al., 2017) and more variable reaction times, slower movements and more movement dissynchrony with an interaction partner (Fulceri et al., 2018).

Besides investigations of alignments of whole body or limb movements, mutual gaze and the establishment of joint attention are of particular interest for INTERpersonal coupling processes (Emery, 2000; Senju and Johnson, 2009). Gaze behavior is the first non-verbal source for the coordination of behavior between newborn and parent and therefore a driving force for the development of non-verbal reciprocity (Feldman, 2007). Empirical evidence for atypical gaze behavior and atypical processing of gaze cues in individuals with ASD is now overwhelming, in particular early aversion of social gaze (Jones and Klin, 2013), altered attention preferences for social cues in form of gaze avoidance (Madipakkam et al., 2017) and less contact or involvement evoked by direct gaze (Schwartz et al., 2010). Gaze idiosyncrasies were already found in children with ASD (Jones and Klin, 2013) and are still present in adults (Schwartz et al., 2010; Georgescu et al., 2013; Madipakkam et al., 2017; Caruana et al., 2018) and are not caused by oculomotor disfunctions (Caruana et al., 2018). In conclusion, empirical evidence from several domains indicate reduced body motion alignment, less anticipation of other persons' kinematics in motor planning as well as atypical social gaze as features of individuals with ASD that contribute to INTERpersonal dissynchrony (see Table 1 for an overview).

**Table 1:** Studies on INTERpersonal synchrony in ASD.

**Study**	***N* (m;f)**	**Age *M*(*SD*)**	**Paradigm**
**OSZILLATION**			
Fitzpatrick et al. (2016)	9 (8;1)	13.7 (1.3)	Pendulum task
Marsh et al. (2013)	8 (6;2)	6.2 (1.2)	Rocking chair task
**BODY ALIGNMENT**			
Fitzpatrick et al. (2017a,b)	45 (39;6)	8.6 (4.8)	Social motor synchronization tasks and cognitive measures
Noel et al. (2018)	12 (8;4)	12.2 (3.8)	Multisensory temporal binding task and MEA
**JOINT ACTION**			
Curioni et al. (2017)	16 (13;3)	26.1 (/)	Grasping objects in social vs. non-social condition
Fulceri et al. (2018)	11 (10;1)	7.8 (1.3)	Joint action task with clear and unclear end point
Gonzalez et al. (2013)	10 (9;1)	32.7 (10.8)	Helping partner by passing objects
Scharoun and Bryden (2016)	14 (9;5)	8.6 (/)	Grasp-to-reach task with experimenter
Studenka et al. (2017)	5 (3;2)	9.8 (/)	Narrative task and motor perspective taking
**SOCIAL GAZE**			
Caruana et al. (2018)	17 (11;6)	26.5 (11.9)	Initiating and responding to joint attention
Jones and Klin (2013)	11 (11;0)	0.2–0.4	Gaze preferences in longitudinal study design
Madipakkam et al. (2017)	14 (8;4)	35.4 (2.3)	Unconscious reactions to direct and averted gaze
Schwartz et al. (2010)	20 (11;9)	39.3 (9.2)	Socioaffective effects of direct gaze

*All studies recruited age-matched control groups*.

## 2. INTRApersonal Dissynchrony in ASD

In their social entrainment model, McGrath and Kelly (1986) consider social interaction in terms of temporal patterns or rhythms in behavior. This model states that endogenous (i.e. individual) rhythms in behavior become temporally aligned in phase and period in the course of interaction. This implies the emergence of systematic temporal patterns of verbal and non-verbal turn-taking during INTERpersonal encounters. Based on this, one can assume that there exist temporal windows of signal production that are critical for communication efficiency and INTERpersonal alignment. From an individual perspective, communication signals are composed of various non-verbal sources (e.g., gaze and gestures). These need to be coordinated with each other and with verbal output to achieve the intended communicative effects. We define INTRApersonal synchrony as the temporal coordination of communication signals in a socially informative manner. In the following, we will review evidence of atypical temporal processing and movement patterns in ASD. We will then introduce the idea that those peculiarities may be related to individuals with ASD missing the assumed temporal windows for producing socially effective communication signals.

### 2.1. Temporal Processing in ASD

Temporal processing of sensory input seems to be altered in individuals with ASD. For instance, in a perceptual simultaneity task individuals with ASD judged the presentation of two visual stimuli to be temporally asynchronous for smaller stimulus onset asynchronies compared to typically developed (TD) control participants (Falter et al., 2012a). Further empirical evidence shows an enhanced temporal parsing of auditory (Jones et al., 2009) and visual events (Falter et al. 2013; but see Isaksson et al. 2018), lower hit rates for the detection of differences in temporal intervals between auditory signals (Falter et al., 2012b), atypical judgment and reproduction of durations (Szelag et al., 2004) and wider multisensory temporal binding windows for simultaneity judgments (Noel et al., 2018). All of these findings support the notion of atypical temporal processing in ASD, possibly associated with a detail-focused, less holistic cognitive style as postulated in the ‘Weak Central Coherence Theory’ of autism (Happé and Frith, 2006). Atypical temporal processing also manifests in higher level processes, such as the subjective experience of time. Allman et al. (2014) in this context argue that stereotypical behavior patterns and behavioral routines serve the structuring of subjective time experience in ASD which compensates for atypical internal timing functions. In line with that are results of a high tendency in ASD to rely on self-structured routines and repetitive behavior to control bottom-up perceptual input, thereby generating experiences of timelessness or “flow” (Vogel et al., 2018a,b).

Atypical temporal processing in ASD most likely influences behavior as well, given that sensorimotor frameworks propose feedback loops of sensory and motor systems (Wolpert et al., 2003; Torres et al., 2013). In this line, Gowen and Miall (2005) found atypical motor timing in an ASD sample, namely faster and more variable responses in a finger tapping task and results were replicated by Isaksson et al. (2018). In addition to this evidence for altered motor timing, behavioral research underpins the assumption that individuals with ASD exhibit atypical movement patterns, as shown in the following literature.

### 2.2. Motor Production in ASD

Clinically, “clumsiness” in motor production is a major feature of autism (Asperger, 1944). Although still a secondary criterion for diagnosis, Parma and de Marchena (2015) argue that atypical motor patterns in ASD need to be further investigated as they occur across the spectrum and may constitute a possible diagnostic marker. In this context a study by Anzulewicz et al. (2016) successfully discriminated children with ASD from TD children by a machine learning algorithm that deployed motor variables from a gaming task with a touch screen. Children with ASD exhibited significantly faster movements with peculiar pressure patterns. Other studies further highlight jerky limb movements (Cook et al., 2013), atypical gait (Barrow et al., 2011; Kindregan et al., 2015; Dufek et al., 2017; Eggleston et al., 2017), enhanced postural sway (Gowen and Miall, 2005; Doumas et al., 2016) and enhanced variability in motor output (Brincker and Torres, 2013; Gowen and Hamilton, 2013; Parma and de Marchena, 2015; Kaur et al., 2018). A meta-analysis by Fournier et al. (2010) included 41 studies on motor coordination, motor impairment, arm movement, gait, or postural stability. They found a significant effect indicating weaker motor performance in ASD individuals, independent from symptom severity. A review by Gowen and Hamilton (2013) systematically inspected approaches on motor abilities in ASD on the background of a computational model that postulates intermediate cognitive steps of motor processing. The authors suggest poorer integration of sensory input for motor planning as well as increased variability in motor output or “motor noise” as integral characteristics in ASD.

Taken together, there is cumulative evidence for atypical movement patterns in terms of reduced coordination and greater variability in motor production. Together with evidence for peculiarities in motor timing (Gowen and Miall, 2005; Isaksson et al., 2018), movement aberrations may influence INTERpersonal communication because communicative signals as motor acts dissociate from typical signal production with respect to temporal emergence.

### 2.3. INTRApersonal Dissynchrony in Interactions

The Autism Diagnostic Observation Schedule (ADOS) as a standard diagnostoc tool targets the coordination of communication channels as a symptom of ASD (Lord et al., 2000). Regarding social contexts there exists research in atypical gesture production in individuals with ASD. In a study by de Marchena and Eigsti (2010), the authors counted gesture usage and coded types of gestures in a narrative task with ASD and TD adolescents. They found no differences in frequency and the kind of gesture used but atypical timing of gestures related to co-occurring speech led to reduced ratings of communication quality in naive observers. In another study on gesture usage in infants with ASD, Colgan et al. (2006) also found no differences in the frequency of gestures, but infants with ASD showed a reduced variety of gestures compared to TD control participants. That is in line with findings of less complexity in non-verbal behavior found by Noel et al. (2018). These results indicate that it is not the quantity of communicative signals that leads to the known communication difficulties but the quality of signals and how they fit in the interactional flow. In the social context it is noteworthy to mention, that the temporal thresholds of perceptual simultaneity (that indicated enhanced temporal parsing of sensory events) in ASD were significantly correlated with difficulties in the communications domain, especially when difficulties were assessed with items encompassing the use of communicative gestures and social imitation (Falter et al., 2012b). Isaksson et al. (2018) likewise found an association of enhanced temporal parsing and symptom severity in communication and social interaction. Noel et al. (2018) further demonstrated that multisensory temporal binding windows correlated with INTERpersonal synchrony in TD participants but not in participants with ASD, indicating distinctive associations in the multisensory temporal domain.

Those findings imply that temporal processing in ASD may be associated with the reduced INTERpersonal alignments. If this association is mediated by atypical social signal timing, is targeted by our proposed perspective on INTRApersonal dissynchrony in ASD.

## 3. Perspective on Future Research

The temporal model of social interactions by McGrath and Kelly (1986) implies that synchronous alignments require mutual responsiveness and coordinated signal production. Individuals with ASD exhibit atypical temporal processing and motor patterns, what most likely disrupts the emergence or maintenance of systematic INTERpersonal coupling. In line with that, we argue that future research needs to extend findings of deviant motor timing (Gowen and Miall, 2005; Barakova and Chonnaparamutt, 2009; Isaksson et al., 2018) to the domain of socially expressive behavior and investigate the impact of INTRApersonal dissynchrony on interactions.

Therefore we suggest approaches on INTRApersonal dissynchrony should pursue two consecutive goals. First, the aim is to quantify temporal deviations in communication behavior in ASD and to find critical temporal windows of INTRApersonal synchronous signal production. State-of-the-art techniques, such as motion capture, eye tracking and video tracking should be used to assess time series of communication behavior. This allows the investigation of peculiarities of signal timing in multiple communication contexts. Combining such techniques makes it possible to assess the temporal coordination of separate signal sources (e.g., gaze, gestures, facial expressions, speech) in terms of relational signal onsets, durations and end points. Thereby one may gain insights into the temporal composition of individual signal streams. On the background of findings of enhanced motor variability (Brincker and Torres, 2013; Gowen and Hamilton, 2013; Kaur et al., 2018) the investigation of measures of dispersion in ASD samples will be of particular interest. Furthermore, implementing perceptual timing tasks may lead to insights in functional relations of temporal processing and social motor timing in ASD. Thus, the questions if timing of communicative channels is affected by a general sensory timing deficit or by social contexts or both can be addressed by comparing task performance in social and non-social tasks of varying sensory complexity. With regard to the neurophysiological framework of predictive coding, it would be highly interesting to investigate, if INTRApersonal dissynchrony also manifests in EEG patterns with social cues produced by individuals with ASD possibly missing predictive timing windows (Arnal and Giraud, 2012). As physical arousal and stress may be enhanced in social tasks in ASD participants, further assessment of heart rate and skin conductance constitute important covariates.

A subsequent goal would be to analyze the perception of idiosyncratic communication patterns, here targeting causal effects of INTRApersonal dissynchrony on INTERpersonal outcomes. Therefore, motion capture data should be used to animate virtual characters in order to create ecologically valid and standardized stimulus material for perception studies (Bente and Krämer, 2002; Georgescu et al., 2014; Pan and Hamilton, 2018). By assessing impression, evaluation and recognition of altered signal production in ASD, one may draw causal conclusions for deficits in social interactions. Dependent variables should be included in such perception studies that are critical for the quality of the produced signal, e.g., communication efficiency and measures of INTERpersonal bonding, e.g., likeability. Creating an “autistic avatar” would allow experimental manipulation of movement parameters under high experimental control. It is of great relevance to illuminate the perspective of the interaction partner to fully understand developmental pathways and resulting communication deficits. There is evidence that TD participants show poorer performance in decoding expressive movements generated by individuals with ASD, indicating reciprocal lack of mentalization (Edey et al., 2016). On presentation of short video clips or still frames of individuals with ASD, independent raters judged individuals with ASD less favorably and reported less motivation to socially approach them (Sasson et al., 2017). An avatar that exhibits specific autistic movement patterns could therefore be employed for research into reciprocal effects of INTERpersonal dissynchrony as well as for training of staff to improve interaction with patients.

This approach on INTRApersonal dissynchrony in ASD potentially has further implications for diagnosis and therapy. In this context, measures of INTRApersonal communication signal coordination could serve as implicit measures that can be used for diagnostic purposes. Implicit diagnostic tools are strongly needed to account for symptomatical heterogeneity in ASD. Subjective observational tools are based on clinical observations or self-report with limited objectivity, especially in adults given behavioral adjustment throughout their lives. Time series data of motion patterns may be used for diagnostic purposes, e.g. supported by machine learning (Georgescu et al., 2019). Our recent work shows that automatized classification of ASD from non-ASD is possible on the mere basis of motion energy assessed using video analysis (ibid.). Specifically motion capture data of INTRApersonal movement parameters is likely to further increase classification power due to richer data retrieval.

An INTRApersonal approach has conceivable implications for the field of robotics in autism research as temporal parameters of signal production may inform models of interactive robotic behavior.

Recent research of human-robot interaction (HRI) with children with ASD revealed positive effects, as robots attract attention and elicit novel behavior while social complexity can be controlled for (Duquette et al., 2008; Scassellati et al., 2012; Srinivasan et al., 2016). The AURORA project (AUtonomous RObotic platform as a Remedial tool for children with Autism) used the robot “Robota,” which resembles a human doll and is able to exhibit interactive movements via video-, speech-, and motion-tracking (Dautenhahn and Billard, 2002). The project showed that “Robota” could serve a mediating role for eliciting joint attention in triadic human-human-robot interactions and elicited spontaneous imitation behavior (Dautenhahn and Werry, 2004; Robins et al., 2004, 2005), thereby potentially reinforcing social skills.

Amplifying joint attention via HRI is of great potential for endorsing social engagement and reciprocity in children with ASD, but the literature is not yet fully convincing. In their study, Anzalone et al. (2014) found that children with ASD were less responsive to joint attention initiatives by the social robot “Nao” and both groups responded less to the robot compared to a human therapist. Another approach investigated interactions of four children with “Nao” and again found mixed results, including facilitated joint attention only for one child (Tapus et al., 2012). Possibly, the design of the social robot with respect to its anthropomorphism may be highly important for eliciting and reinforcing social interactive behavior in children with ASD, for example a realistic eye design in joint attention paradigms (Admoni and Scassellati, 2017; Luria et al., 2018). However, the target of the intervention is not yet properly defined.

Our perspective suggests that individuals with ASD exhibit social interaction in different ways (e.g. peculiar temporal parameters of communicative signal production). Socially interactive robots generally need to be able to recognize communicative signals and exhibit appropriate reactions (Breazeal et al., 2016). Thus, models of robotic behavior could be adjusted to temporal parameters of signal production in ASD in order to reinforce reciprocity, similar to computational approaches in Admoni and Scassellati (2014) or Barakova and Chonnaparamutt (2009). Such an adjustment may enhance compliance and responsiveness of individuals with ASD toward the robotic interaction partner. Furthermore, given that Gowen and Hamilton (2013) suggest intact motor learning in ASD, parameters of typical signal timing may be used for robotic interventions that aim to train proper timing in communicative signal coordination, thereby providing a possible quantitative outcome measure of treatment success. Building upon findings of the AURORA project, the creation of HRI scenarios in which human-like robots serve as interactive tutors for training specific communicative skills (e.g. joint attention) are promising. Creating game-based robot interactions that prompt spontaneous imitation of properly coordinated signals could be a great opportunity to support children in their development of non-verbal skills.

There exist a number of aspects that need to be considered when planning approaches on INTRApersonal dissycnhrony in ASD. One potentially confounding factor when measuring INTRApersonal synchrony lies in the distinction between spontaneously and voluntarily produced behavior, as different cognitive processes are thought to underlie these processes (Frith and Frith, 2008; Torres et al., 2013). Thus, future studies should investigate how INTRApersonal dissynchrony differs under the impact of explicit instructions or implicit and natural task conditions. Furthermore, highly standardized study designs that strictly control sensory surroundings are crucial for studying INTRApersonal synchronization, given deviant sensory processing may contribute to behavioral variability in ASD.

Further research should broaden this approach to other psychiatric disorders that entail INTRApersonal coordination peculiarities like schizophrenia (Walther et al., 2015) or depression (Schrijvers et al., 2008). But especially for ASD, we think that a perspective on INTRApersonal dissynchrony is fundamentally relevant for understanding INTERpersonal difficulties. A quantification of temporally atypical coordination of communication signals in ASD is an important explanatory approach that potentially informs diagnosis as well as intervention programs.

## Author Contributions

In accordance with theoretical discussions with CF-W and KV, CB wrote the first manuscript version. AG contributed literature and theoretical ideas. All authors read and modified the manuscript several times. All authors listed have made a substantial, direct and intellectual contribution to the work, and approved it for publication.

### Conflict of Interest Statement

The authors declare that the research was conducted in the absence of any commercial or financial relationships that could be construed as a potential conflict of interest.
